# Commentary: “*Prom1* Function in Development, Intestinal Inflammation, and Intestinal Tumorigenesis”

**DOI:** 10.3389/fonc.2015.00091

**Published:** 2015-04-21

**Authors:** Christine A. Fargeas, Edgar Büttner, Denis Corbeil

**Affiliations:** ^1^Tissue Engineering Laboratories (BIOTEC), Technische Universität Dresden, Dresden, Germany

**Keywords:** cancer, CD133, prominin-1, murine model, retinal degeneration, testis

A large body of publications mentioning prominin-1 (Prom1, CD133) is related to its use as a stem and cancer stem cell marker (1). Besides progenitor cells, its expression is widespread throughout terminally differentiated cells as those found in glandular organs (e.g., pancreas) and retina (2). Yet, the amino acid sequence of Prom1 didn’t reveal any motif or potential enzymatic activity that could explain its molecular function (3). Nonetheless, its implication in cellular physiology has been addressed in several cellular systems and animal models. In particular, the effect of *Prom1* gene ablation has been investigated in different tissues and organs (Table 1, top). In the above-mentioned article (4), erroneously qualified by its authors as “the first study to provide an in-depth evaluation of the role and function of *Prom1*”, several features of a new Prom1^-/-^ mouse were highlighted that were not described in previous Prom1-deficient murine models, raising questions about the influence of the genetic backgrounds on the penetrance and variable expressivity. To understand these issues, it is important to have an accurate view on the implication of Prom1 in molecular processes leading to tissue homeostasis and diseases. Unfortunately, valuable information that could potentially enlighten some of the data reported was omitted (4). Moreover, the genetic construct and the characterization of their model (protein expression) were missing, thus preventing direct comparison with previous works. We propose to provide the readers of *Frontiers in Oncology* with a larger context for the observations of Karim et al. and the potential functions of Prom1.

**Table 1:** **Prom1 genetically modified murine models and mutations in human *PROM1* gene**.

Genotype	Disrupting exon Gene ID 19126	Type	Background	Reported phenotype	Reference
**Murine models with genetically modified *prom1* gene**					
*Prom1*^-/-^	Exon 2	Constitutive knockout	Congenic C57BL/6	Disk dysmorphogenesis and photoreceptor degeneration	(5)
			50/50 129/swiss	Reduced branching in mammary gland	(6)
				Enhanced ratio of luminal to basal cell	
			Congenic C57BL/6 JOlaHsd	Normal blood cells pool size	(7)
				Reduced frequencies of growth factor-responsive myeloerythroid precursor cells *in vitro*	
			C57BL/6	Normal proliferation of precursor cells in adult hippocampus subgranular zone Reduced number of new neurons surviving	(8)

*Prom1^lacZ,DTA/lacZ,DTA^*	Exon 2	LacZ knockin	C57BL/6	No abnormal phenotype	(9)
			C57BL/6	Photoreceptor cell degeneration	(10)
				Difficulty in breeding	
			C57BL/6 xCBA/NSlc	Photoreceptor cell degeneration with slower progression	

*Prom1^lacZ,DTA/+^; CreER^TM^*	Exon 2	Conditional ablation of Prom1-expressing cells upon DTA expression by tamoxifen-induced Cre mediated recombination	C57BL/6 xCBAxSJL	Abnormal nervous system morphology	(9)
				Reduced body weight	
				Walking abnormality (cerebellum functional defect)	

*Prom1^C-L/C-L^*	Exon 2 (first ATG)	CreER LacZ knockin	C57BL/6	No abnormal phenotype	(11)

*Prom1^C-L^/Prom1^+^; Rosa26-YFP; Ctnnb1^+/lox(ex3)^*	Exon 2 (first ATG)	Conditional activation of endogenous Wnt signaling in Prom1-expressing cells by tamoxifen-induced Cre mediated recombination and lineage tracing	–	Increased intestinal adenocarcinoma incidence after tamoxifen administration	(11)

*Prom1^rd19^*	Exon 9	Spontaneous knockout (premature STOP codon; K269X)	BXD83/RwwJ	Retinal degeneration	
				Abnormal retinal blood vessel morphology	
					http://www.informatics.jax.org/allele/MGI:5605699

*Prom1^lacZ/+^*	Exon 3–8	Expression reporter	C57BL/6J	No phenotype analysis, expression assay	(12)

*Prom1^-mCherry-IRES-CreERT2^; Rosa26-LacZ*	Disruption of the STOP codon	Fusion protein Lineage tracing	–	No phenotype analysis	(13)

*Prom1*^-/-^	?		129SvEv	Mature obesity	(4)
				Moderate degree of germinal arrest Increase in fasting blood glucose	

**Mutations in human *prom1* gene that affect its open reading frame**					
c.1878delG	Frame-shift from codon 614^a^ onward causing premature STOP codon after the addition of 12 amino acids; G614fs12X			Autosomal recessive retinitis pigmentosa Polydactyly	(14)

c.1726C > T	Premature STOP codon; Q576X			Severe forms of rod-cone degeneration consistent with retinitis pigmentosa	(15)

c.1349_1350insT	Frame-shift from codon 452 onward causing a premature STOP codon after the addition of 12 amino acids (exon12) Y452fs12X			Autosomal recessive cone-rod dystrophy	(16)

c.1117C > T	Single amino acid substitution; R373C			Autosomal dominant macular dystrophy	(17, 18)
				Possible impairment in endothelial progenitor cell functionality	

c.869delG	Frame-shift from codon 289 onward causing a premature STOP codon after the addition of one amino acid; S289fs1X			Retinitis pigmentosa and macula degeneration	(19)

c.442A > T	Premature STOP codon; K148X			Retinitis pigmentosa	(20)

*^a^According to the sequence of Prominin-1.s2 splice variant [for nomenclature of splice variants see Ref. (21)]. Mutations are indicated according to the proposed nomenclature (22)*.

*?, not described*.

In humans, several mutations in *PROM1* gene affecting the open reading frame were found to be associated with retinitis pigmentosa, macular degeneration, and cone-rod dystrophy (Table 1, bottom). Despite the extended expression of PROM1, little phenotypical effects were observed beyond the visual system. Extensive clinical analysis of patients carrying PROM1 R373C mutation suggested that endothelial function could be affected despite apparently normal levels of endothelial progenitor cells (23). Neuro-imaging revealed many small lesions in the cerebral white matter in three patients. The expression of Prom1 in myelinating oligodendrocytes might be relevant in this context (24). Some carriers of the R373C mutation showed memory disturbance and impairment in measured executive functions, suggesting that the lack of functional PROM1 may not solely affect photoreceptor cells. Yet, the penetrance was variable, calling for larger studies to confirm these findings (23).

In the first description of Prom-1^-/-^ mice, they were reported as viable and fertile, with a normal lifespan and no obvious abnormalities upon macroscopic inspection and histological analysis of various organs other than a progressive photoreceptor degeneration leading to complete loss of vision (5), thus constituting a mouse model of the human diseases. These observations have been recently confirmed in an independent Prom1-deficient murine model that also revealed that this degeneration was light dependent, consistent with what is observed in Stargardt’s disease patients (10). Interestingly, variations in the genetic background influenced the progression of photoreceptor cell degeneration (10). In addition to engineered animal models, a spontaneous knockout mouse (*Prom1^rd19^*) carrying single point mutation in *Prom1* gene was reported with retinal degeneration and abnormal retinal blood vessel morphology (Table 1). Moreover, using a transgenic knock-in mouse carrying the human dominant R373C mutation, Yang et al. could demonstrate the structural involvement of Prom1 in photoreceptor morphogenesis (17). As a cholesterol-binding protein associated with membrane microdomains, Prom1 could provide a proper membrane lipid composition and synchronize various steps in the outer segment biogenesis. Whether the new mouse line of Karim et al. also suffers visual impairment is not known but it appears to present several features not yet described (4).

Notably, they noted compromised spermatogenesis in “some Prom1^-/-^ males” (4). It would be interesting to know what proportion is affected, since Prom1 is expressed in the male reproductive tract of mouse and human (25, 26), and is detected in mouse spermatozoa found in the testes and the epididymis (25, 27, 28). Similarly, the mature obesity in Prom1^-/-^ mice reported by Karim et al. based on ≈15% increase in body weight over a 13-week period compared to wild type is different from earlier studies (Table 1), and may reflect the influence of genetic background on the permeability to metabolic disorders. In fact, in a conditional ablation model, tamoxifen-induced ablation of Prom1-expressing cells caused body weight loss (9). Studies have pointed to the potential involvement of Prom1 in cellular metabolism in rat myotubes and mouse pancreatic islets with contrasting findings (29, 30), and an increase in Prom1 expression in young mice with induced obesity has been reported (31). In other respects, PROM1 was shown to promote glucose uptake in a human hepatocellular carcinoma cell line (32). Karim et al. also indicated an increase in blood glucose levels and therefore suggested a link between Prom1 and pancreatic function (4). Although the metabolic features of Prom1^-/-^ mice need to be more thoroughly investigated (e.g., insulinemia, gain in adipose tissue, glucose tolerance), it is worth mentioning that Prom1 labels fetal mouse and human islet progenitor cells (33, 34) and was used for isolation of pancreatic ductal progenitor cells (35). PROM1 is expressed in ductal cells of the exocrine component of adult pancreas (36–38).

The novel Prom1^-/-^ mouse was maintained in 129SvEv, a less phenotypically characterized background than the frequent C57BL/6 and might be more permissive to the expression of these characters. Therefore, whether the differences in various Prom1-deficient mice are related to strain, the different background (39), environmental factors, or the construct would require further studies. Yet, the various cellular activities with which Prom1 has been associated are in line with our early suggestion that Prom1 can act as regulator in the organization and functionality of plasmalemma protrusions (40). Hence, its absence may cause alterations in cellular adhesion and/or signaling pathways. Although the phenotypic consequences of Prom1 defect seem to be limited, despite a broad tissue expression, to a restricted number of organs especially those devoid of prominin-2 (e.g., retina and testes) (3, 27), a careful examination over longer periods of time may uncover the expression of subtle changes in functionality in specific tissues.

## Conflict of Interest Statement

The authors declare that the research was conducted in the absence of any commercial or financial relationships that could be construed as a potential conflict of interest.
